# Evaluation of Probable Sarcopenia’s Prevalence in Hospitalized Geriatric Patients Using Ishii’s Score

**DOI:** 10.7759/cureus.49158

**Published:** 2023-11-21

**Authors:** Ana Cavalheiro, Sara Afonso, Marta Silva, Nuno Ramalhão, João Machado, Sandra Magalhães

**Affiliations:** 1 Physical Medicine and Rehabilitation, Centro Hospitalar Universitário de Santo António, Porto, PRT

**Keywords:** mortality, functional dependency, hospitalized patients, sarcopenia, geriatrics

## Abstract

Background: Sarcopenia is defined as a progressive loss of skeletal muscle mass and strength related to age and comorbidities. Worldwide sarcopenia’s prevalence varies between 10-40%, being associated with functional impairment, lower quality of life, and higher mortality. Sarcopenia can be estimated based on age, calf circumference, and handgrip strength (Ishii’s formula). Early diagnosis is essential because treatment with nutritional support and rehabilitation programs can prevent complications. The aim of the study was to assess the prevalence of probable sarcopenia in hospitalized patients using Ishii’s* score* and possible associated risk factors.

Methods: We developed an observational prospective study in a medicine inpatient ward of a Central Hospital. We applied Ishii’s formula to the patients admitted to the medical ward in December 2021 and January 2022. Patients should be aged 60 or above and able to collaborate with the tests. One year later, we analyzed re-hospitalization and mortality rates. Patients with edema of the lower limbs, who were not able to follow instructions, and who were admitted exclusively for symptomatic treatment were excluded.

Results: Our final sample was 49 patients (55% males, mean age 78 ± 8.88 years). Only one patient had a previous diagnosis of sarcopenia. Estimated sarcopenia´s prevalence was 73.5% N=36), higher in men and people with three or more comorbidities. In the sarcopenic group, 77% had some degree of functional dependency and positive markers for malnutrition. After one year of follow-up, we found a higher mortality rate in the sarcopenic group (44.4% against 7.6%) and a higher number of re-hospitalizations (1.03 hospitalizations per patient, against 0.31).

Conclusions: Our data showed that the prevalence of probable sarcopenia is high, but this pathology is still underdiagnosed. Traditional diagnosis is complex in some hospital settings and a simple tool such as Ishii’s score can help to improve diagnostic rates. We suggest screening all patients at admission to provide early rehabilitation and nutritional support.

## Introduction

Sarcopenia is a public health problem with an estimated global prevalence of 10-40% [1-4]. Its prevalence is higher in older adults, especially in those who are institutionalized [1,4]. This pathology includes a progressive and generalized loss of muscle mass and strength. It is primarily associated with aging (primary sarcopenia), but a sedentary lifestyle can also contribute to sarcopenia’s development [1,5]. Comorbidities such as cardiovascular or respiratory disease, malnutrition, alcohol consumption, smoking, or stress can originate chronic inflammation, promoting a catabolic state that contributes to the loss of muscle (secondary sarcopenia) [5].

According to European Working Group on Sarcopenia in Older People (EWGSOP) guidelines from 2010, sarcopenia consists of a decrease in skeletal muscle mass (adjusted for height), a decrease in muscle strength (hand grip strength), and/or low physical performance (evaluated by six-minute walk test or timed up and go test) [1].

The identification of reduced muscle mass involves the execution of specific diagnostic tests such as Dual-energy X-ray Absorptiometry (DEXA) scan, computer tomography, magnetic resonance, bioimpedance evaluation, and rectus femoris thickness by ultrasonography, but in some settings performing these tests can be challenging due to economical and logistic constraints [1].

Sarcopenia has a major socio-economic impact, contributing to increased risk of falls and fractures, prolonged hospitalizations, lower quality of life, and higher mortality rates [2,6,7]. Investigation and concern about sarcopenia are rising and since 2017 it has been recognized as a muscle disease with a diagnosis code from the 11th International Classification of Diseases, but the exact prevalence is still unknown [8].

The reported prevalence rates vary across different populations. Some authors reported rates between 0.4 and 9.3% in older adults from a Canadian community and can reach 20% in males over 80 years old [9-11]. In terms of geriatric hospitalized patients, data is limited but a recent study of patients admitted to acute geriatrics and geriatric rehabilitation showed a sarcopenia prevalence of 22% [12].

In recent updates from EWGSOP, it was emphasized that there was a shift toward muscle strength, making low muscle strength a key characteristic of sarcopenia [1,2]. This led to the birth of a new term, “probable sarcopenia,” that is defined as a decrease in muscle strength and its presence makes sarcopenia’s diagnosis very likely [2]. In this update, it was also suggested that poor physical performance can be a marker for severe sarcopenia [2]. If “probable sarcopenia” is present, an investigation should be completed and treatment should be started due to the risk of further complications [2]. 

As said before, identifying this disease as soon as possible is essential not only to prevent complications but also because current evidence supports that an adequate rehabilitation program based on exercise and nutrition monitoring can slow down the progression [1].

In spite of the recent efforts to make the diagnosis process easier, data on sarcopenia’s prevalence is still very heterogeneous due to various operational definitions, different cut-off points among populations, and difficulty in accessing complex diagnostic tools such as DEXA scan.

Recently, different sarcopenia screening tools have been created and tested, such as the SARC-F questionnaire, which is a tool that is validated for different populations [13,14]. In Japan, Ishii and his colleagues developed a formula based on age, calf circumference (CC), and handgrip strength (HS) that predicts the probability of probable sarcopenia [15]. This screening tool is described as easy to perform and requires simple material, a millimeter-graded tape, and a hand dynamometer [15]. This score is already validated in the Japanese population and it is a valid predictor for all-cause mortality in hospitalized Chinese older adults [14].

The aim of our study was to evaluate the prevalence of probable sarcopenia using Ishii’s score, associated risk factors, and one-year mortality in patients admitted to an internal medicine ward at a Central Hospital.

## Materials and methods

An observational prospective study was conducted in an internal medicine inpatient ward of a tertiary hospital in Porto, Portugal. The study protocol was approved by the Research Ethics Committee of the Hospital (IRB 2021.090). Written informed consent was obtained from all participants. All methods in this study were in accordance with relevant regulations and guidelines.

Study population

We included all patients who were admitted in the medical ward, during December 2021 and January 2022. The inclusion criteria were patients aged 60 or above and who were able to collaborate with the tests. Patients with edema of the lower limbs, who were not able to follow instructions, and who were admitted exclusively for symptomatic treatment were excluded.

Definition of probable sarcopenia

We followed the recommendation of the European Working Group on Sarcopenia in Older People (EWGSOP) for the definition of probable sarcopenia [1]. The proposed diagnostic criteria required the presence of low muscle strength.

Measurement of handgrip strength and calf circumference

We measured handgrip strength (HS) and calf circumference (CC) and then, we applied Ishii’s formula to calculate probable sarcopenia’s predicting score [15].

We measured the CC with the patient in a supine position, on the dominant leg, left knee flexed and the calf placed at a 90º angle to the thigh, using a millimetre‐graded tape to the nearest 0.1 cm [15].

The HS was measured with a Jamar Hydraulic handheld dynamometer (Performance Health, the United States) to the nearest 0.1 kg. Participants were positioned with the elbow flexed at a 110° angle, the wrist in a neutral position, and the interphalangeal joint of the index finger at a 90° angle. Bilateral hand grips were measured three times and the highest value was recorded [8].

The formula to calculate Ishii scores is as follows: in men, 0.62 × (age − 64) − 3.09 × (HS − 50) −4.64 × (CC − 42); in women, 0.80 × (age − 64) − 5.09 × (HS − 34) − 3.28 × (CC − 42). The cutoff points for defining sarcopenia were ≥105 for men and ≥120 for women [15].

Measuring functional dependency

We asked the patients directly about their habits and their ability to perform daily living activities one month before admission (bathing, dressing, eating, and walking). With that information, we classified them with a four-point Likert scale as “autonomous” (no need for help), “minimal dependent” (minimal help in one of the tasks), “partial dependent” (moderate help in less than three tasks) or “totally dependent” (needed intense help in all tasks).

Data collection

We collected data from medical records and interviews referring to gender, weight, height, smoking habits, and alcohol consumption. We also questioned the patients or family members about their previous functional capacity. By reviewing online files, we collected data about usual medication and comorbidities (hypertension, diabetes, ischemic heart disease, chronic obstructive pulmonary disease, kidney disease, osteoarthritis, osteoporosis, cancer, gastrointestinal or liver disease). We also collected data from blood tests concerning levels of albumin, iron, vitamin D, folic acid, and calcium.

Statistical analysis

The descriptive variables are presented as absolute numbers and frequencies for categorical variables. For normally distributed variables we used the mean ± standard deviation (SD). The Pearson chi-squared test was used for categorical variables and a one-way ANOVA for continuous variables.

Data analysis was performed using Statistical Package for Social Sciences (SPSS) version 28.0 (IBM Corp., Armonk, NY), with p<0,05 indicating statistical significance.

We applied univariate and multivariate Cox proportional-hazard models to calculate the hazard ratio (HR) and 95% confidence interval (CI) for factors associated with sarcopenia as 3 or more comorbidities, albumin level, BMI, and functional status.

## Results

Population’s characteristics

We initially recruited a total of 130 participants, but only 49 patients met the inclusion criteria. Eighty-one patients were excluded because of severe cognitive impairment (N=32), leg edema (N=33), or because they were in exclusively symptomatic treatment (N=16).

Our sample included 49 patients with a mean age of 78 years old, with a total of 27 men (55%) and 22 women (45%). Concerning functional capacity, 17 were autonomous in daily activities (35%), 11 minimal dependent (22%), 15 partial dependent (31%), and 6 totally dependent (12%). Table 1 shows the baseline characteristics of participants according to sarcopenia status.

**Table 1:** Baseline characteristics of participants BMI: body mass index; COPD: chronic obstructive pulmonary disease; HF: heart failure; CKD: chronic kidney disease; HT: hypertension; DM2: diabetes mellitus type 2

Characteristics	
Age (Mean ± SD)	78 ± 8.9
Male sex % (N)	55% (27)
Functional capacity % (N)	34.7% autonomous (17)
	22.4% minimal dependent (11)
	30.6% partial dependent (15)
	12.2% totally dependent (6)
BMI (Mean ± SD)	22.5 ± 5.96
Comorbidities	
COPD	44.9% (22)
HF	59.2% (29)
CKD	22.4% (11)
HT	79.6% (39)
DM2	42.9% (21)
Smokers	28.6% (14)
Osteoarthritis	16.3% (8)
Osteoporosis	10.2% (5)

Prevalence of probable sarcopenia

Based on Ishii’s formula, 36 participants had a predictive score for sarcopenia, making a prevalence of 73.5%. The probable sarcopenia group had a higher number of older patients and from male sex with a statistically significant result (Table 2).

**Table 2:** Relationship between sarcopenic classification and BMI, dependency, hospitalization, and mortality rate BMI: body mass index

Characteristic	Probable sarcopenia N=36	Non-sarcopenic N=13	p-value
Age (Mean ± SD)	80.1 ± 8,1	72.2 ± 8.8	<0.05
Male sex % (N)	63.9% (23)	31% (4)	<0.05
Functional capacity % (N)	25% autonomous (9)	62% autonomous (8)	<0.05
	22% minimal dependent (8)	23% minimal dependent (3)	
	36% partial dependent (13)	15% partial dependent (2)	
	17% totally dependent (6)	0% totally dependent (0)	
BMI (Mean ± SD)	23.5 ± 5.4	28.2 ± 5.2	<0.05
Low albumin levels	55.6% (20)	15.4% (2)	<0.05
Number of hospitalizations in last year (Mean ± SD)	1.03 ± 1.04	0.31 ± 0.41	0.053
Mortality rate at 1 year (N)	44.4% (16)	7.6% (1)	<0.05

Malnutrition

We aimed to analyze malnutrition status based on total proteins and albumin levels. In total, we found low albumin levels in 22 patients (44.9%) but in 11 patients this data was not available (22.4%). In terms of albumin levels, patients with probable sarcopenia were more likely to have lower values (HR=6.0, p<0.05). We did not analyze data about blood levels of total proteins, iron, vitamin D, folic acid, and calcium because, in the majority of patients, these values were not tested during hospital stay.

Body mass index 

On average, the included patients had a BMI between the normal range (22.5 ± 5.96). We found five patients with lower BMI (10.2%) and all had a predictive score for sarcopenia. The percentage of overweight was higher in the non-sarcopenic group (69.2%) compared with the percentage in the sarcopenic group (27.7%). 

Comorbidities 

In the sarcopenic group, we found a higher number of comorbidities per person (72.2% had three or more comorbidities) compared with the non-sarcopenic group (53.8% had three or more comorbidities).

Functional dependency

We also detected that patients with an Ishii score predictive of sarcopenia were more likely to have a higher number of comorbidities (OR=2.23, p=0.16) and lower functional capacity without statistical significance (OR=6.15, p=0.06).

Mortality and hospitalization rate

After one year of enrolment, we analyzed mortality and hospitalization rates. In the group with probable sarcopenia, we found a mortality rate of 44.4% against 7.6% in the non-sarcopenic group. In terms of hospitalization rate, we found a mean of 1.03 hospitalizations per patient in the sarcopenic group, against 0.31 hospitalizations per patient in the group with a non-predictive score for sarcopenia (Table 2).

## Discussion

Our data show probable sarcopenia has a high prevalence among hospitalized people (prevalence 73.5%), especially in older men. If we compare with reported data on sarcopenia’s prevalence, we find our number much higher than the previous reports; Smoliner et al. described a prevalence of 24.6% in a Swiss population and Martone et al. reported 14.6% in an Italian one [3,4]. If we analyze probable sarcopenia prevalence, data is scarce, but Sobestianky et al. reported 73% prevalence in community-dwelling elderly men, although this study only included men [10]. Actually, Ishii’s score is a predictive score for sarcopenia based on the reduction of muscle mass and strength which is more related to probable sarcopenia definition.

In a recent meta-analysis, including community-dwelling older adults, the application of EWGSOP and Foundation for the National Institutes of Health Sarcopenia Project (FNIH) definitions led to a high variability in sarcopenia’s prevalence (from 0 to 37% and 3 to 73%, respectively) [10]. The authors explained these variations due to definition and measurement techniques, whether sarcopenia was measured using a single measure of muscle mass or a combined measure of muscle mass and muscle strength and/or physical function [10]. This proves the heterogeneity of data depending on the definition used and the type of population analyzed. 

In terms of gender prevalence, there are controversial results. Gao et al. studied the associated risk factors of sarcopenia and they found a higher risk of sarcopenia in men (OR 1.50 without statistical significance) [16]. On the other hand, Zhang et al. reported that female sex was significantly associated with a higher risk for sarcopenia (OR 0.31) [17].

We also detected a higher prevalence of low albumin levels and higher functional dependency among the group with a positive Ishii score. In all of the previously mentioned studies, a positive association between malnutrition and sarcopenia was also described [2-4,18].

We aimed to analyze malnutrition status based on total proteins and albumin levels but much of this data was missing. Although we did not have strong data about the malnutrition status of our patients, it is possible that this comorbidity is present. So our suggestion is to screen at admission albumin, total proteins, and ions such as calcium, iron, folic acid, and vitamins for all patients above 65 years old or with risk factors for sarcopenia.

In our study, we found that individuals with an Ishii score predictive of sarcopenia had higher mortality and hospitalization rates at one year of follow-up. Previous studies have also found similar results. De Buyser et al. followed men of age 74 or more from a semi-rural community in Belgium, and they concluded that low grip strength, low BMI, and low functional status were associated with mortality [19]. On the other hand, Yang et al. conducted a prospective study in acute care wards of a hospital in China for 3 years and they found that the mortality of sarcopenic participants was significantly increased compared with non‐sarcopenic participants (40.8% vs. 17.1%, respectively) [20]. All of these factors have a negative impact on the individual but also at an economic level. Goates et al. analyzed the impact of hospitalizations in American adults with sarcopenia and found they had higher odds for hospitalization and a total estimated cost of USD 40.4 billion per year [21].

Even though there is a growing awareness and interest in this pathology, sarcopenia is still a misdiagnosis. In our study, only one patient had a prior diagnosis of sarcopenia, although our findings suggest that it might be present in over 70% of our patients. In theory, healthcare professionals should be more alert to identify this pathology and there are multiple diagnostic tests available but this has not been translated into clinical practice. In Portuguese hospitals, we found it difficult to perform tests such as DEXA scan or Bioelectrical Impedance Analysis. It’s important to raise medical awareness but it’s also essential to implement easy and valid screening tests to identify probable sarcopenia as early as possible. During this investigation, we found Ishii’s score a simple and inexpensive tool that can be easily applied in a hospital setting. Our experience is that Ishii’s score is an easy and simple tool to apply in a hospital setting. We used a metric tape and a portable analogic dynamometer, which allowed us to directly measure CC and HS in patients’ hospital wards. We then calculated individual Ishii scores with the Excel tool, which was also very easy to perform.

Previous studies with this tool showed good sensitivity and specificity, indicating that Ishii’s score can be a quick form to identify individuals at risk [22]. To our knowledge, this tool is only validated in Japanese and Chinese populations [14,23], but there is a need to perform the validation of this tool in European populations [22].

Limitations

Some limitations need to be addressed. We included a small number of participants which limits the generalization of our results. We also could not evaluate performance status and classify sarcopenia’s severity, due to logistic constraints in the ward. In terms of assessing functional dependence, we used a classification based on a Likert scale that is in use in our service, but this might limit the reproducibility of our study. Additionally, some data from blood tests were missing because they were not tested during the hospital admission. In the future, it would be important to implement a systematic screening for all patients including the analysis of some parameters such as albumin, total protein levels, ions, and vitamins. 

Additionally, as said before, Ishii’s score is not yet validated in European populations, so the actual score might not be totally adequate for our population. Looking ahead, we will take forward our investigation and try to validate Ishii’s formula in the Portuguese population.

## Conclusions

Sarcopenia is a highly prevalent disease that affects essentially older persons with frailty syndrome leading to an increasing risk for hospitalization and death. This condition leads to personal, social, and economic burdens so early diagnosis and accurate treatment are mandatory. Ishii’s score was an easy and simple tool to use in a hospital setting to screen for probable sarcopenia prevalence. We look forward to progressing our investigation and validating Ishii’s formula in the Portuguese population.
